# Using Targeted Interventions to Strengthen Water, Sanitation, Hygiene and Infection prevention to control the 2023 Cholera outbreak

**DOI:** 10.1017/ash.2025.372

**Published:** 2025-09-24

**Authors:** Shillah Nakato, Martin Esagala, Bonny Kintu, Daniel Bulwadda

**Affiliations:** 1 Infectious Diseases Institute, Makerere University; 2 IDI; 3 Ministry of Health

## Abstract

**Introduction:** Cholera disproportionately affects vulnerable populations, with outbreaks occurring mostly in locations with limited access to clean water, and adequate sanitation. Uganda experiences cholera outbreaks almost yearly, with the July -August 2023 outbreak in the central and eastern regions registering over 30 confirmed cases and 5 deaths. Cholera outbreaks are anticipated to increase especially during the rainy season, straining Uganda’s healthcare system. We implemented multimodal IPC/WaSH interventions within the affected communities and their respective Health Facilities (HFs) to control the transmission and prepare HFs to identify and manage cholera cases. **Methods:** Between July and August 2023, we deployed multimodal targeted interventions to deliver WaSH/ IPC interventions to health facilities (HFs) and communities in eight most affected sub-counties. We initially conducted WaSH/IPC assessments using the Ministry of health (MoH) scorecard at 31 high risk HF. At HFs, interventions focused on addressing gaps identified from the assessments including training of health workers (n=325) to improve hand hygiene, environmental cleaning, and waste elimination practices. We provided WaSH/IPC supplies to further improve practices. The assessments were repeated after the interventions, and we compared findings using a paired t-test. At community level, we trained 25 VHTs to conduct hand hygiene promotion at 6000 households within the case clustered villages. we also sensitized 500 community members at highly trafficked community sites such as fishing and markets. **Results:** At baseline, the mean WaSH/IPC score at HFs was 45% (Standard deviation (SD)=17.7), which improved to 72% (SD=9.9; p) **Conclusion:** Given the improved WaSH/IPC practices at HFs and absence of secondary transmission to other sub counties, the targeted interventions show promise for use in future cholera outbreaks in similar settings. However, with the anticipated increase in cholera outbreaks due to climate change, and the ongoing shortage of cholera vaccines, interventions that address the root causes of cholera outbreaks are increasingly important.

